# Item recommendation and quantum correlation on multiple datasets

**DOI:** 10.1038/s41598-026-48508-0

**Published:** 2026-05-22

**Authors:** P. Bhaskaran, S. Prasanna

**Affiliations:** School of Computer Science Engineering and Information Systems, Vellore Institute of Technology, Vellore, 632014 TamilNadu India

**Keywords:** Quantum recommender system, Quantum physics, Entanglement, Quantum circuits, Mathematics and computing, Physics

## Abstract

Recommender systems are crucial for customizing user experiences across diverse sectors such as e-commerce and entertainment. Traditional correlation methods have been employed to forecast user preferences; however, they frequently prove inadequate when addressing intricate, high-dimensional datasets. Quantum computing presents a new approach for enhancing correlation computations, potentially resulting in more precise recommendations. This study seeks to address and compare the efficiency of classical and quantum correlation techniques in recommender systems utilizing four distinct datasets: Supermarket Sales, IMDB Top 250 movies, MovieLens 10k, and BigBasket products. The Item Recommendation and Quantum Correlation (IRQC) method, makes use of parameterized quantum circuits with rotation gates and entanglement. The experimental methodology comprised the utilization of both classical and quantum correlation approaches, evaluating their efficacy through critical metrics including mean absolute error (MAE) and root mean squared error (RMSE). The results demonstrated that quantum correlations consistently surpassed classical correlations across all datasets. The proposed Quantum Correlation approach obtains lower mean absolute errors of 0.99, 0.30, 0.90, and 0.92 in BigBasket, Supermarket Sales, IMDB Top 250 Movies, and MovieLens 10K datasets, respectively, than 1.20, 1.48, 1.10, and 1.00 with the classical methods. This study underlines the potential of quantum computing in machine learning applications, notably for boosting recommendation systems.

## Introduction

In today’s digital landscape, the vast amount of information offers users an overwhelming variety of choices, whether it’s selecting a movie to watch, a book to read, or a product to purchase. With this explosion of possibilities, the desire for individualized advice has never been greater. Recommendation systems have developed as vital tools, using large amounts of data to deliver personalized suggestions based on individual tastes. Traditional recommender systems rely on traditional algorithms and data processing techniques to provide recommendations based on user preferences and item features^1^. However, these systems generally struggle to represent the complicated interactions and interdependence between numerous variables. Classical correlation is a conventional technique used in data analysis to measure the degree and direction of linear relationships between variables^2^. While classical correlation is useful for assessing linear relationships, it may struggle to capture non-linear correlations and complex interactions among variables, especially in highly-dimensional data spaces. Quantum Correlations introduce a novel technique by combining principles from quantum computing and machine learning^3^. This unique technique promises to boost suggestion accuracy by exploring non-linear correlations and complicated interactions.

In recommender systems, classical correlation methods are extensively employed across many fields to know the relationship between one to another variable. The Pearson correlation coefficient is a prevalent approach for determining the linear relationship between two continuous variables^4^. This metric is frequently utilized in item recommendation systems to assess the similarity between user preferences or item characteristics. Pearson correlation emphasizes linear relationships, although alternative methods such as Spearman’s rank correlation and Kendall’s tau are employed to identify non-linear relationships and ranked data. In item recommendation systems, correlation measures have been essential in enhancing recommendation quality by recognizing items or individuals with similar behavior patterns.Even though classic correlation techniques exhibit obstacles, particularly when addressing complicated interactions or multi-dimensional datasets, they frequently ignore complex, non-linear patterns in high-dimensional data, perhaps resulting in unsatisfactory recommendations. The increasing demand for precise and advanced recommendation systems underscores the necessity for alternative methodologies, such as quantum correlation techniques, that allow for deeper and more complex correlations.

A fundamental understanding of quantum computing is necessary to grasp the concept of quantum correlations. Quantum computing operates on qubits, the basic units of quantum information, which can exist in a state of superposition, representing both 0 and 1 simultaneously^5^. Furthermore, quantum entanglement enables qubits to exhibit strong correlations across large distances, enabling a more sophisticated representation of user preferences and item attributes^6^.

The core idea of quantum processing is the utilization of quantum circuits formed by utilizing quantum gates to manipulate qubits. This paper explores the integration of Item Recommendation and Quantum Correlation, presenting a novel approach that incorporates quantum principles into the recommendation process. By using quantum circuits and recommendation approaches, the ultimate goal is to improve the accuracy of item recommendations. This will result in more personalized and interesting experiences for users on e-commerce platforms.

The IRQC framework delivers the following key contributions:

- The importance of IRQC is that it can find good correlations that regular similarity measures miss, especially when dealing with high-dimensional or sparse data.
- Results on various real-world datasets in experiments show IRQC obtains lower MAE and RMSE than classical baselines consistently.

The primary objective of this research is to build a novel approach for recommending top-correlated products or movies utilizing quantum correlations. This research presents a novel quantum correlation strategy for item recommendations that consistently surpasses classical correlation methods across various datasets. In addition to that, the implementation leverages quantum circuits specifically for correlation-based item suggestions, demonstrating their usefulness in capturing complicated interactions in the data that conventional approaches typically fail to perform effectively. To achieve this, Pennylane, an open-source software platform built for quantum machine learning and computing, is used^7^. Section 2 presents previous research work done by other researchers, Section 3 proposes a method for item recommendation and quantum correlation, Section 4 presents results and discussion, and Section 5 concludes the research.

## Related works

Classical correlation methods have been fundamental to item recommendation systems, particularly through collaborative filtering, content-based filtering, and hybrid approaches. Early work by Hayashi focused on refining item-item similarity metrics for recommender systems, suggesting improvements over the widely-used Pearson correlation coefficient^8^. Gong et al. utilized the Pearson correlation coefficient to determine the linear correlation between variables, combining both linear and nonlinear measures to provide a comprehensive assessment of variable relationships^9^. Several other studies have used Pearson correlation to enhance item recommendations. Rahman et al. leveraged Pearson correlation to assess the linear relationship between item ratings, facilitating movie recommendations based on user preferences^10^. Similarly, Mana et al. applied Pearson correlation to measure the linear relationship between user ratings, identifying similar preferences among users to recommend relevant items^11^. Gadekalu et al. introduced a weighted Pearson similarity, which improves the accuracy of item recommendations by enhancing the correlation between items, leading to lower Mean Absolute Error (MAE) and Root Mean Square Error (RMSE)^12^.

In more advanced applications, Fan et al. proposed Learning Correlation Information for Multi-label Feature Selection (LCIFS), which uses the Pearson coefficient to address feature redundancy, achieving strong performance on multi-label datasets^13^. Vijayvargiya et al. incorporated Pearson correlation-based graphs into a Graph Neural Network (GNN), creating a multistage classification technique that demonstrated high accuracy in classifying healthy and abnormal knee conditions^14^. Furthermore, Shoaib et al. developed a hybrid model that employed Pearson correlation and outperformed the K-Nearest Neighbor algorithm in the precision of movie recommendations^15^.

When dealing with non-parametric data, the Spearman rank correlation is commonly employed. Othman et al. provide a robust method for assessing Spearman rank correlation without assuming a normal distribution^16^. Zhang et al. used the Spearman rank correlation coefficient to assess user similarity based on ranked ratings, effectively capturing nonlinear relationships and handling outliers in item recommendations^17^. Jiang et al. developed the SPESI algorithm, which uses the Spearman coefficient along with class-driven precision to enhance feature selection and improve computational efficiency^18^.Other researchers have explored alternative rank correlation measures. Koike et al. compared asymptotic variance measures of concordance, including Spearman’s rho, Blomqvist’s beta, and Kendall’s tau, finding that Kendall’s tau exhibited superior performance in terms of asymptotic variance^19^. Pedroche et al. employed the Kendall correlation coefficient to improve neighbor selection in collaborative filtering, leading to enhanced item recommendations by identifying users with similar preferences^20^. In parallel, Sankaran and Ganesh introduced a similarity-based deep learning model (SDLM) for an automatic movie recommendation system, achieving efficient results across various performance metrics^21^.

While classical correlation methods have proven effective, they are often limited in capturing complex, high-dimensional relationships. Quantum Correlations, in contrast, offer new dimensions for improving recommendation systems. Pilato et al. explored Quantum Correlations through the Standard Quantum-Based (SQB) similarity, leveraging Hilbert space vectors to measure item correlations, leading to enhanced recommendation quality^22^. Batra et al. extended this work by focusing on quantum entropy and discord to better capture user preferences and improve recommendation accuracy^23^. Building on these ideas, Wang et al. modeled user preferences using quantum many-body wave functions, effectively capturing complex interactions to improve recommendation accuracy^24^. Shajilal et al. introduced a novel correlation measure, Entropic Accord (EA), which demonstrated robustness as a correlation metric in quantum-based frameworks^25^. Satoori et al. investigate key entanglement variables, including concurrence, quantum discord, and entanglement entropy, offering insights into the dynamics of strongly correlated quantum systems near critical points^26^. Lastly, Vesperini et al. focus on graph states and the role of quantum correlators, like Entanglement Distance and Pauli matrices, in achieving improved entanglement and connectivity properties in quantum recommendation systems^27^.

Recent advancements have led to the emergence of hybrid quantum-classical recommender systems, which synergistically combine the computational power of quantum algorithms with the stability and adaptability of classical methods. Li et al. proposed a quantum-enhanced collaborative filtering algorithm that leverages quantum computing principles within a hybrid framework, significantly improving recommendation accuracy and speed, particularly on large-scale datasets^28^.Raychaudhuri et al. introduced a hybrid recommendation model that integrates collaborative filtering with demographic information and quantum-enhanced algorithms, effectively addressing data sparsity and the cold-start problem^29^. Ouedrhiri et al. proposed a hybrid model that combines quantum K-means clustering with classical matrix completion, enhancing both prediction accuracy and computational efficiency^30^. Sinha et al. explored the use of Quantum Neural Networks (QNNs) for movie recommendations, demonstrating that their hybrid neural architecture outperforms traditional models in capturing complex user-item interactions^31^. Gao et al. presented a novel quantum recommender system that can be viewed as a quantum version of classical matrix reconstruction algorithms and introduced a quantum variant of the alternating least squares (ALS) algorithm, enabling the derivation of factor matrices in quantum state form and facilitating the reconstruction of the original rating matrix^32^. Gronlund et al. examined the theoretical limits of quantum approach in recommendation tasks, showing a provable exponential separation between quantum and quantum-inspired classical algorithms in solving sparse linear systems, while highlighting the practical challenges of sustaining quantum benefits in hybrid implementations^33^. Additionally, Roberto Campos focused on hybrid algorithms for search optimization and the evaluation of quantum algorithms in chemistry, emphasizing their modular nature and broad applicability in quantum artificial intelligence and related domains^34^. Collectively, these works underscore the growing potential of hybrid quantum-classical recommender systems to transcend the limitations of traditional approaches by effectively leveraging contemporary quantum technologies.

IRQC consists of an angle-based data encoding, controlled entangling and rotational gates are used explicitly to encode inter-item and user item dependencies in the quantum circuit. The expectation values of measurement operators are used to obtain the correlation score, which captures effects of quantum interference and entanglement at the scale of quantum correlation measures and is not explicitly used to do in previous hybrid quantum recommenders. Furthermore, in comparison to QNN based techniques to approximate classical learning pipelines, IRQC is a non-learning circuit-driven correlation model, which provides lightweight and expressive quantum technique. The nature of this design is that the IRQC is essentially distinct in terms of its encoding strategy, definition of correlation, and inference procedure, which can be characterized as a novel quantum correlation strategy.

## Methods

The focus of this analysis is on recent developments in the field of quantum recommender systems versus classical recommender systems. Quantum Correlation can be used to characterize the relationship between distinct features or attributes in a movie dataset. In this situation, each feature is similar to a quantum state. The correlation between characteristics is calculated to better understand how they relate to and influence one another. For instance, you may examine the correlation between a movie’s title and its target audience’s interest rating. Likewise, the product examines the correlation between a product name and its target rating based on user preferences.

The research work is classified in two ways: one is based on the traditional correlation of the recommender system technique used, and another is based on the Quantum Correlation of the recommender system. Pennylane libraries are used for the Quantum Correlation method.

### Classical correlation

In classical correlation, the three correlations, such as the Pearson correlation coefficient, the Spearman correlation, and Kendall tau, are compared, and most of the datasets provide a good correlation for Pearson, so it is considered to be compared with quantum correlation. Table 1 depicts the comparison of various classical correlation methods.The top correlated item calculated with Pearson, Spearman, and Kendall coefficients on four datasets-Supermarket Sales, BigBasket, IMDb, and MovieLens 10k-identifying domain-specific trends. On the Supermarket Sales dataset, the comparatively low correlation values indicate the random and heterogeneous nature of item selection at retail purchases. The BigBasket data set has perfect correlations for several item pairs, which tend to be purchased together, implying high co-occurrence patterns characteristic of online grocery shopping. Films like Amelie and Vertigo in the IMDb dataset exhibit perfect correlation with Ikiru, demonstrating regular user patterns for classic or critically rated films and MovieLens 10k; high correlations occur among movies of the same genre or subject, affirming the efficiency of correlation measures in identifying user-consistent patterns. These findings support the application of correlation-based methods in uncovering significant item relations in various domains, forming the basis for comparing their quantum-enhanced counterparts. Among these three classical correlation methods, such as Pearson, Kendall, and Spearman, the Pearson coefficient method performs better for Supermarket Sales, IMDB Top 250 Movies,MovieLens 10k and BigBasket Product. So this is taken into account when comparing it with quantum correlation.

**Table 1:** Classical correlation value of Top N Item recommendations.

**Dataset &** **Input**	**Output of the Top N classical correlated item**		
	**Pearson**	**Spearman**	**Kendall**
SS & Health and beauty	Food and beverages: **0.147704**	Food and beverages : 0.124226	Food and beverages : 0.085714
	Sports and travel: 0.030548	Fashion accessories :0.054749	Fashion accessories : 0.055152
	Electronic accessories : −0.019326	Sports and travel : 0.025339	Sports and travel : 0.016471
	Home and lifestyle : −0.021837	Home and lifestyle: −0.008980	Home and lifestyle : −0.000816
	Fashion accessories: −0.043420	Electronic accessories: −0.043200	Electronic accessories:−0.016591
BB & Tamarind/Hunisehannu	Cleaning Cloth - Micro Fiber, G130018 : 1.0	Stainless Steel Lunch Box/Tiffin Set - Blue, BB 575 2 : 1.0	Stainless Steel Lunch Box/Tiffin Set - Blue, BB 575 2 : 1.0
	Basmati Rice/Basmati Akki - Sehat Mogra : 1.0	Airtight Lock-N-Pack Plastic Container - Transparent : 1.0	Airtight Lock-N-Pack Plastic Container - Transparent : 1.0
	Spunky and Funky Avatar Perfume : 1.0	Eyeconic Kajal : 1.0	Eyeconic Kajal : 1.0
	Coffee Sprinkles - Silky Hazelnut : 1.0	Door/Floor Mat - Multi Surplus : 1.0	Door/Floor Mat - Multi Surplus : 1.0
	5 Meter Flexible Wire Link 1089 : 1.0	DoorMat - Cotton, Assorted Colour : 1.0	DoorMat - Cotton, Assorted Colour : 1.0
IMDB & Ikiru	Amélie : 1.0	Singin’ in the Rain : 1.0	Singin’ in the Rain : 1.0
	Vertigo : 1.0	Eternal Sunshine of the Spotless Mind :1.0	Eternal Sunshine of the Spotless Mind :1.0
	Inglourious Basterds: 1.0	Scarface : 1.0	Scarface : 1.0
	Lawrence of Arabia : 1.0	Good Will Hunting : 1.0	Good Will Hunting : 1.0
	To Kill a Mockingbird : 1.0	The Apartment : 1.0	The Apartment : 1.0
ML10k & Heat(1995)	Philadelphia (1993) : 0.686060	Léon: The Professional (a.k.a. The Professional) (Léon) (1994) : 0.559943	Léon: The Professional (a.k.a. The Professional) (Léon) (1994) : 0.479559
	Slumdog Millionaire (2008) : 0.662325	Shaun of the Dead (2004) : 0.521436	Philadelphia (1993) : 0.464136
	Léon: The Professional (a.k.a. The Professional) (Léon) (1994) : 0.651800	Philadelphia (1993) : 0.518827	Casino (1995) : 0.435388
	Bourne Ultimatum, The (2007) : 0.611546	Army of Darkness (1993) : 0.509918	Shaun of the Dead (2004) : 0.431416
	Aliens (1986) : 0.597448	Miss Congeniality (2000) : 0.509366	Army of Darkness (1993) : 0.419740

#### Pearson

The Pearson correlation coefficient is used as a correlation technique to assess the linear correlation between two variables. The function computes the magnitude and orientation of the linear correlation between two variables, namely the unit prices of several items. The Pearson correlation coefficient spans a range of −1 to 1, with a value of 1 denoting a flawless positive linear association, −1 denoting a flawless negative linear association, and 0 denoting the absence of any linear relationship between the variables.

The Pearson correlation coefficient, denoted by PC, measures the linear relationship between two continuous variables.The variables p1 and p2, and their respective means as $\bar{p1}$ and $\bar{p2}$ the Pearson correlation coefficient can be calculated using the following formula:

1 $$\begin{aligned} \text {PC} = \frac{\sum _{i=1}^{n} (p1_i - \bar{p1})(p2_i - \bar{p2})}{\sqrt{\sum _{i=1}^{n} (p1_i - \bar{p1})^2 \sum _{i=1}^{n} (p2_i - \bar{p2})^2}} \end{aligned}$$

where,

$p1_i$ and $p2_i$ are the $i^{th}$ observations of variables p1 and p2, respectively,

$\bar{p1}$ and $\bar{p2}$ are the means of variables p1 and p2, respectively and

n is the number of observations.

Algorithm 1 depicts the classical correlation using Pearson correlation. In this, the feature matrix is made through a pivot-table representation on the basis of the main numerical interaction in every dataset. In the case of retail datasets like Supermarket Sales and BigBasket, the feature matrix will be constructed with the level of customer rating as the row, the product as the column, and price as the numerical values. In the case of the movie datasets like MovieLens and IMDB, a pivot-table representation is used to create a feature matrix in the form of a user-movie rating list, with rows being the user ID, columns being the movie, and the observed rating forming the matrix element. The matrices are based on pivots to give unified input to both the classical correlation and quantum correlation approaches to ensure transparent and reproducible evaluation across all datasets.

**Algorithm 1:**
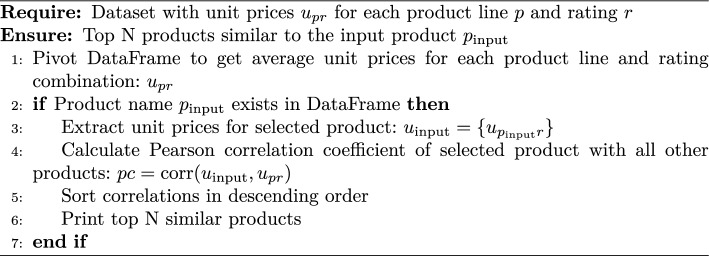
Item Recommendation for classical correlation.

#### Spearman

The Spearman correlation coefficient (SC) of the two variables p1 and p2 with ranks $R_{p1}$ and $R_{p2}$, respectively, is computed as:

2 $$\begin{aligned} \text {SC} = 1 - \frac{{6 \sum _{i=1}^{n} (R_{p1_i} - R_{p2_i})^2}}{{n(n^2 - 1)}} \end{aligned}$$

where,

- *n* is the number of observations (ranked pairs), and

- $R_{p1_i}$ and $R_{p2_i}$ are the ranks of the $i^{th}$ observation for variables *p*1 and *p*2, respectively.

The value ranges between −1 and 1, where 1 indicates a perfect positive relationship, −1 indicates a perfect negative relationship, and 0 indicates no relationship between variables.

#### Kendall

The Kendall rank correlation coefficient (KC) for two variables p1 and p2 with ranks Rp1 and Rp2 is computed as:

3 $$\begin{aligned} \text {KC} = \frac{{\text {{number of concordant pairs}} - \text {{number of discordant pairs}}}}{{\text {{total number of pairs}}}} \end{aligned}$$

where,

-The number of concordant pairs is the number of pairs of items where the rankings agree in their ordering

-the number of discordant pairs is the number of pairs of items where the rankings disagree in their ordering,and

-the total number of pairs is the total number of pairs of items being compared.

A Kendall rank correlation coefficient of 1 indicates perfect agreement between the rankings of items, −1 indicates perfect disagreement, and 0 indicates no association between the rankings.

### Item recommendation and quantum correlation (IRQC)

**Fig. 1:**
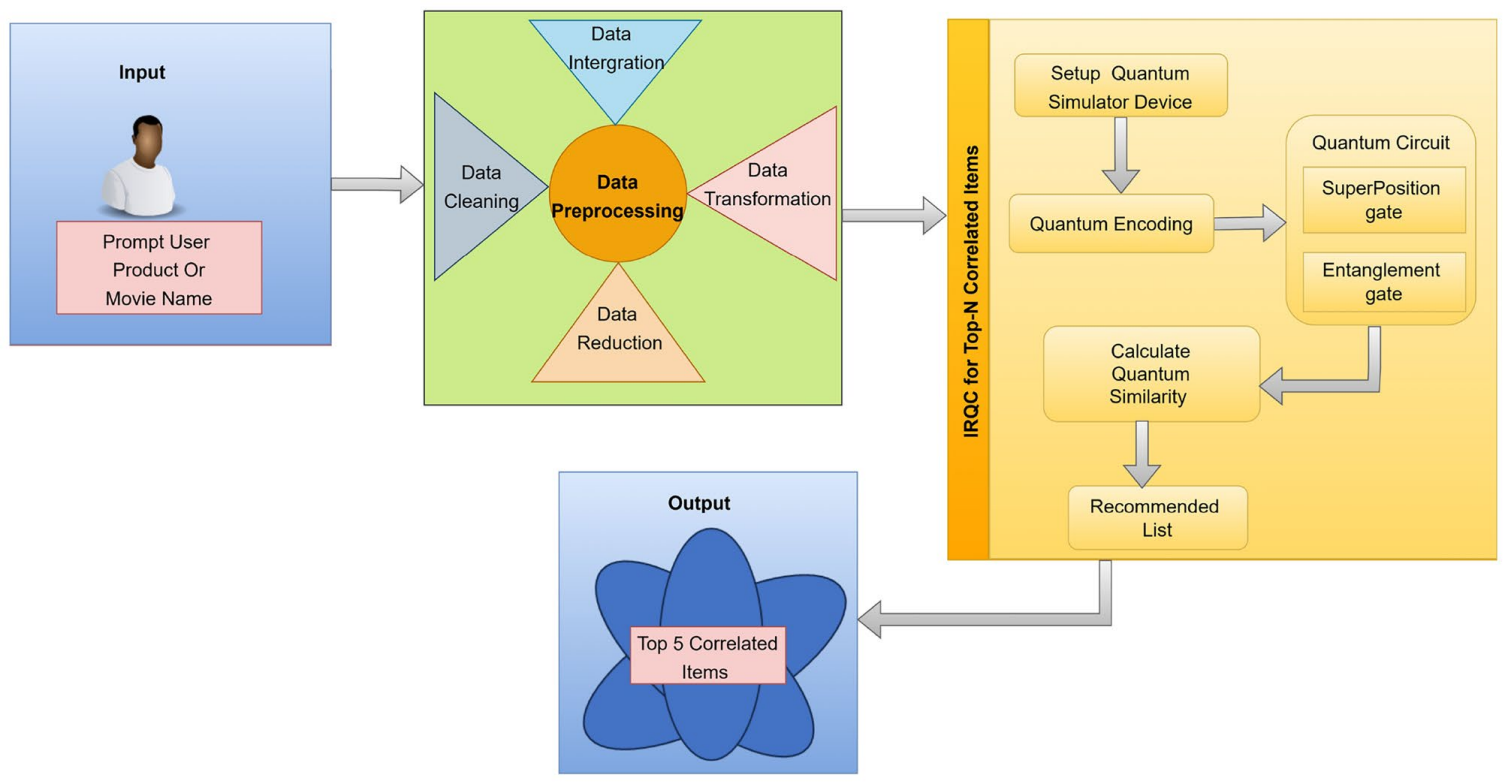
System architecture of item recommendation and quantum correlation (IRQC).

The proposed method shown in Figure 1 shows the Item Recommendation and Quantum Correlation to recommend a product or movie as an output based on Quantum Correlation. This work introduces a new framework known as the item recommendation and quantum correlation (IRQC) to improve the precision of the recommendations model. Most classic recommender systems are based on classical correlation metrics like Pearson, Spearman, or cosine similarity. These methods work well in some cases but lack efficiency when used with sparse user-item matrices, cold-start issues, or non-linear patterns of user preferences. Quantum computing provides a qualitatively different model that can handle complicated relationships among users and items using superposition and entanglement.

In the IRQC method, classical data are encoded as quantum states so that user preferences can be embedded into a Hilbert space. The novelty in IRQC comes from the fact that it employs rotational gates to build a parameterized quantum circuit that encodes user and item rating data. Rotational gates provide the flexibility to represent user preferences as quantum states by mapping classical ratings to rotation angles. This rotational encoding approach overcomes the limitation of fixed gate circuits by providing adjustable quantum states, which can respond to the variability of user preferences. In addition, CNOT and CZ gates are used consciously to introduce entanglement in quantum form, which captures interdependencies between users and items that are hard to describe using classical correlation measures.

After data encoding and entanglement, quantum measurements in the computational basis are executed to obtain quantum state probabilities. These results are mapped to a quantum similarity score that encompasses subtle correlations between user-item pairs. The similarity matrix derived from the IRQC circuit is finally used to predict and generate top-N recommendations.

In this work quantum correlation is a definition of the expectation value of a global Pauli observable on a parametrized entangled quantum state. Consider a variational circuit, the correlation score is computed as $\langle \psi (\boldsymbol{\theta }) | Z_1 \otimes \cdots \otimes Z_n | \psi (\boldsymbol{\theta }) \rangle$, where the parameters encode latent interactions and the entangling gates capture higher-order dependencies.Unlike the classical Pearson correlation which measures linear pairwise dependence in the euclidean space, analogous to quantum correlation measures non-linear, multiple dependences enabled by entanglement in Hilbert space. The quantum correlation approach proposed not only enhances recommendation accuracy but also provides a new paradigm for incorporating the concepts of quantum computing into recommender systems.

The IRQC framework suggested in this work is applied to an ideal noiseless quantum simulator (PennyLane default.qubit), thus, the findings in this work are not to be taken as an indication of quantum advantage in either computational speedup or hardware efficiency. Furthermore, it is better defined as a quantum-inspired similarity mapping scheme, in which classical user-item interactions are represented in a high-dimensional Hilbert space by quantum circuits. Computationally, classical correlation algorithms like Pearson correlation can compute correlation between items in O(n) time, and the IRQC algorithm needs quantum circuit simulation with a cost of O$(2^n)$, n represent number of qubit comprising of state preparation and expectation measurement, which brings further overhead to simulation. As a result, the present implementation does not have a runtime benefit compared to classical approaches. The value of this work, therefore, is not to show quantum speedup, but rather to understand how similarity learning in a recommender system can be speed up by quantum circuit-based transformations.

The first block of IRQC involves initializing a quantum simulator device with‘n’ qubits. The number of qubits depends on the complexity of the problem being solved. The next block involves defining a quantum circuit function called quantum-corr that receives circuit parameters and product name as input. The circuit parameters are encoded into qubits using AngleEmbedding. Next feed into Hadamard gates with n qubits, Further rotational gates such as RX and RY gates are applied based on the circuit parameters, followed by a CZ and CNOT gate between qubits i and i+1. The expectation value of the PauliZ operator on qubits and it is measured, and the correlation is returned.

The next block involves initializing an empty list to store item correlations. For each data point in the Dataset Frame, random circuit parameters are generated, and the Quantum Correlation is calculated using quantum-corr. The retrieved item name and Quantum Correlation are then appended to the list. Finally, this block involves arranging the item’s correlations in decreasing order and picking the top 5 correlated items also it involves measuring the evaluation metrics, such as MAE, and RMSE, and displaying them on the screen.

### Quantum circuit

Quantum circuits consist of quantum gates, which are the basic building parts that carry out operations on qubits. These gates may perform different operations like as flipping the state of a qubit, providing entanglement between qubits, and operating quantum transformations like the Hadamard gate, CNOT gate, and others.Quantum circuits are utilized to execute quantum algorithms, which leverage the properties of quantum mechanics to solve specific computational problems with greater efficiency compared to classical algorithms.

**Fig. 2:**
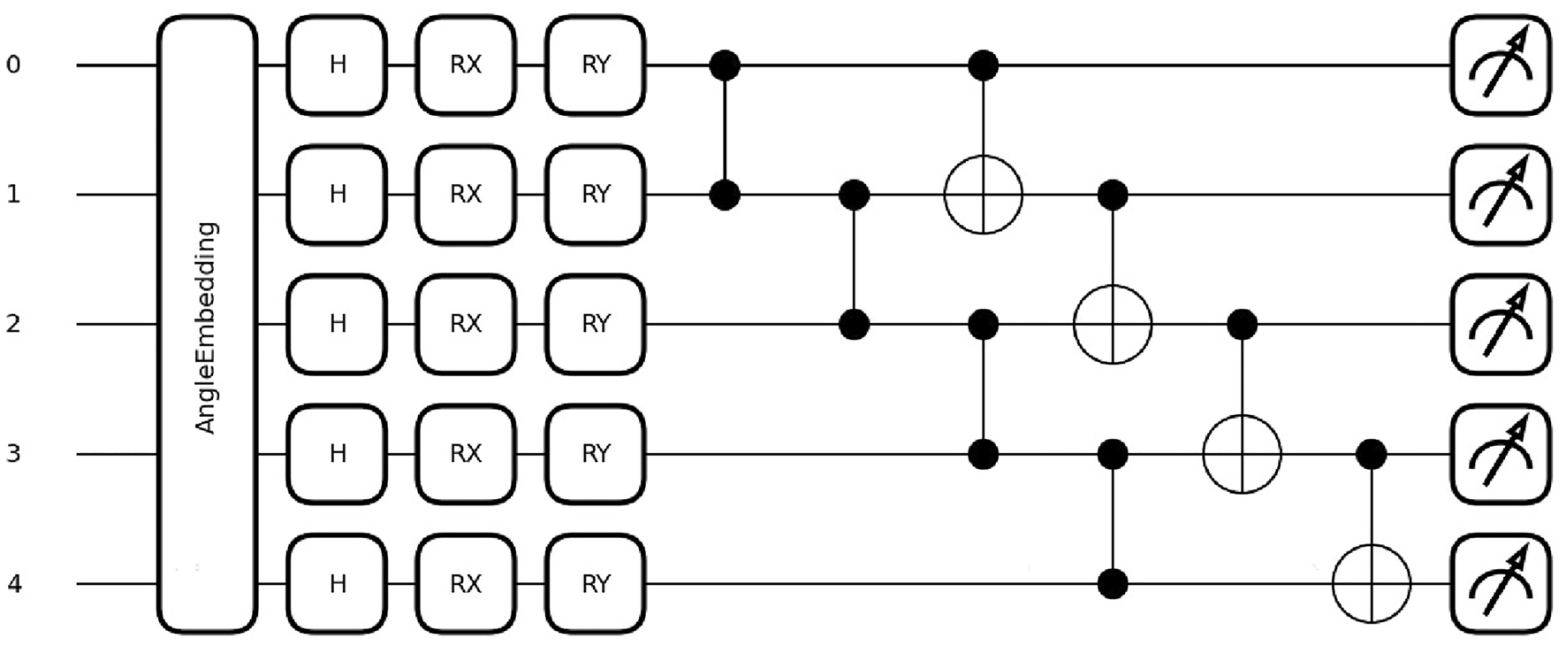
Quantum circuit architecture of the proposed IRQC model.

Figure 2 illustrates the quantum circuit employed in the proposed IRQC technique. The circuit consists of angle embedding, Hadamard gates, rotation gates (RX and RY), controlled-Z gates, CNOT gates, and Pauli-Z operators for measurement. In the first step, classical input angles are encoded into quantum states using angle embedding, followed by Hadamard gates to generate superposition. Subsequently, RX and RY rotation gates are applied to each qubit, after which entanglement is introduced through controlled-Z gates. The qubits are then further entangled using CNOT gates. Finally, the expectation value of the combined Pauli-Z operators across all qubits is measured to obtain the Quantum Correlation.

The quantum circuit used in this research utilizes a five-qubit system simulated on the default.qubit backend of PennyLane, an ideal noiseless quantum simulator. The five-qubit design choice was selected because of the fact that it corresponds to the dimensionality feature of preprocessed interaction features with the representational capacity of quantum circuit. Each qubit represents normalized feature through angle encoding by rotation, and it is expressive enough to calculate correlations and is also practical on simulators. Experimentally, it was found that addition of qubits brings much performance in terms of cost of computation.Classical input information is encoded in quantum states via angle (rotation-based) encoding, namely through the AngleEmbedding template, which projects normalized features onto parameterized rotation gates (RX, RY). The circuit depth is made shallow, involving a single embedding layer followed by parametrized RY rotations and a series of entangling gates (CNOT and CZ) to create quantum correlations between qubits.

In the current implementation, IRQC is a non-learning quantum correlation model, where parameters in the circuit are not optimized, but are data-encoded angles or randomly initialized every time a single correlation is performed.In the present application, the IRQC model is a quantum-inspired random feature mapping model, where the circuit parameters are neither trained nor learned but randomly initialised or generated with the input data. To overcome this instability that may emerge as a result of this design, all experiments are performed over a series of independent experiments with different random initializations. The results that have been reported are related to the mean and the standard deviation of evaluation metrics, which has guaranteed the statistical reliability. This design lets IRQC be a non-linear similarity mapping mechanism similar to classical random feature algorithms.The values of product interaction are then normalized and converted to rotation angles which are encoded with angle-based RX and RY gates in a fixed five-qubit quantum circuit. The circuit is an order of sequence of Hadamard, rotational, controlled Z and CNOT gates to invoke quantum interference and entanglement of the encoded features. The experiments are performed on default.qubit simulator in analytic mode (i.e., infinite-shot execution) of Pennylane and Quantum Correlation is calculated as the expectation value of a product of Pauli-Z operators. This value is directly used to rank correlated products, without any variational training or post-processing, this expectation value which lies in the range between −1 and 1.

#### Angle embedding

The AngleEmbedding template is typically used for encoding classical data into a quantum state. It might be more appropriate to use another template or define custom operations, depending on the specific task.

4 $$\begin{aligned} |\psi _1\rangle = \prod _{i=0}^{n-1} RX(\theta _i){\psi _0}\rangle \end{aligned}$$

#### Hadamard gates

The Hadamard gate H is applied to each qubit.

5 $$\begin{aligned} |{\psi _2} \rangle = \prod _{i=0}^{n-1} H_i |{\psi _1}\rangle \end{aligned}$$

where,

$H_i$ is the Hadamard gate applied to qubit i.

#### Rotation gates

6 $$\begin{aligned} |\psi _3\rangle = \prod _{i=0}^{n-1} RX(\theta _i) RY(\phi _i) |\psi _2\rangle \end{aligned}$$

where,

RX$(\theta _i)$ is a rotation around the X-axis by an angle $\theta _i.$

RY$(\phi _i)$ is a rotation around the Y-axis by an angle $\phi _i$.

#### Controlled-Z gates

This operation applies an entangling CZ gate between adjacent qubits.

7 $$\begin{aligned} |\psi _4\rangle = \prod _{i=0}^{n-2} CZ_{i,i+1} |\psi _3\rangle \end{aligned}$$

where,

$CZ_{i,i+1}$ is the controlled-Z gate between qubits i and i+1.

#### Controlled-not gate

This operation applies an entangling gates, controlled-NOT gate between qubits i and i+1.

8 $$\begin{aligned} |\psi _5\rangle = \prod _{i=0}^{n-2} CNOT_{i,i+1} |\psi _4\rangle \end{aligned}$$

#### Measurement

This operation measures the expectation value of the tensor product of Pauli Z operators on n_qubits.

9 $$\begin{aligned} \langle Z_0 Z_1 \cdots Z_{n-1} \rangle = \langle \psi _5 | \bigotimes _{i=0}^{n-1} Z_i | \psi _5 \rangle \end{aligned}$$

The equation 10 is the final representation of quantum circuit which involve in the IRQC.

10 $$\begin{aligned} \langle Z_0 Z_1 \cdots Z_{n-1} \rangle = \langle \psi _0 | \left( \prod _{i=0}^{n-2} CNOT_{i,i+1} CZ_{i,i+1} \right) \left( \prod _{i=0}^{n-1} RY(\phi _i) RX(\theta _i) H_i RX(\theta _i)\right) \left( \bigotimes _{i=0}^{n-1} Z_i \right) | \psi _0 \rangle \end{aligned}$$

where,

$\langle Z_0 Z_1 \cdots Z_{n-1} \rangle$ represents the entire quantum circuit’s operation.

### Algorithm

The Algorithm 2 illustrates IRQC using PennyLane for recommending items. A quantum circuit is created to calculate correlations between different products based on randomly initialized quantum parameters. The quantum circuit parameters are randomly set to operate as a quantum feature generator as opposed to a trained model. This method is analogous to random feature techniques in traditional machine learning in that it is expressive and non-linear with regards to similarity estimation without optimization bias. Reproducibility through deterministic quantum simulator and fixing the pseudo-random seed ensures the absence of randomness, whereas the stability is promoted through averaging the results of numerous independent executions and presenting the statistical significance metrics. User input is used to select a product, and Quantum Correlations are computed with a five-qubits quantum circuit. These Quantum Correlations are then compared with ground truth correlations to and mean absolute error (MAE) and root mean squared error (RMSE) are calculated as evaluation metrics.In contrast to traditional recommender systems that depend on established similarity metrics (e.g., Pearson, spearman), our suggested method utilizes a quantum variational circuit to represent item correlations inside a non-linear, high-dimensional quantum framework. The algorithm incorporates sale price and user rating data by angle encoding into a 5-qubit quantum circuit, with each product’s feature vector represented by quantum rotational gates (RX, RY). The incorporation of Hadamard, Controlled-Z (CZ), and CNOT gates facilitates quantum entanglement among neighboring qubits, enabling the model to grasp interdependent product linkages that conventional algorithms cannot access. Additionally, the expectation value of the Pauli-Z observable serves as a surrogate for the intensity of quantum correlations. This innovative method captures both individual and pairwise interactions inside the latent product space, facilitating the identification of highly linked goods in a quantum-enhanced similarity framework. This hybrid quantum-classical method provides a novel approach to improving suggestion quality in scenarios of data sparsity and non-linearity, signifying a significant advancement toward practical quantum recommender systems.

**Algorithm 2:**
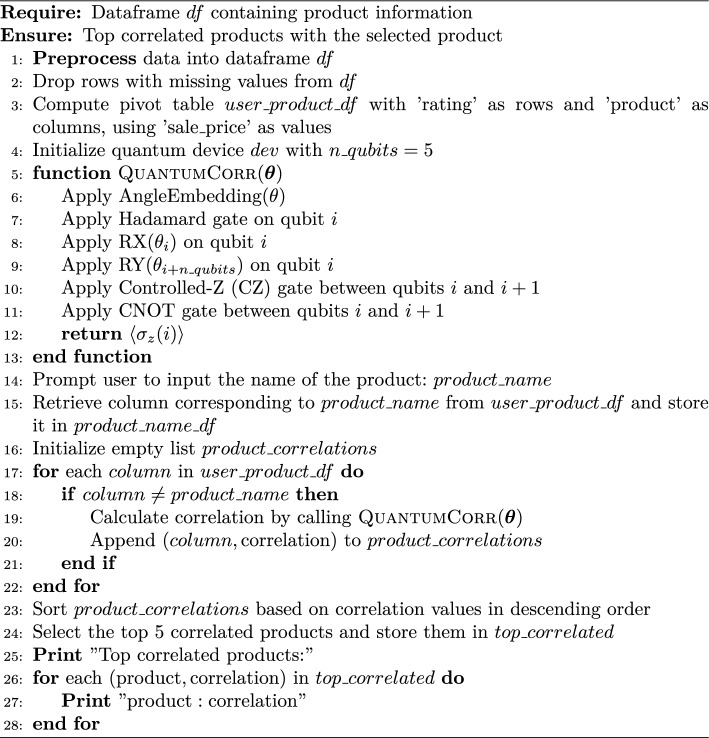
Item Recommendation and Quantum Correlation.

The quantum circuit computes a quantum correlation score of every pair of items, i.e., movies or products, which is obtained as the expectation value of a multi-qubit Pauli observable of Z. These scores are between −1 and 1 to reflect the similarity of items with one another. To calculate them, the quantum correlation scores of the first k items in the list of correlated items are simply compared with the same rows in a classical ground truth correlation matrix calculated with Pearson correlation. The comparison of the Quantum Correlations and the classical correlations is then measured with MAE and RMSE, with the classical correlations being used as reference targets.

### Metrics

Mean absolute error (MAE) and root mean squared error (RMSE) metrics^35^ provide us with information on the accuracy of predictions and the extent to which they deviate from the actual values. Normalized Discounted Cumulative Gain (NDCG) is employed to assess the quality of recommendation lists by taking into account both the relevance and the arrangement of items. Each metric provides valuable insights into different aspects of model performance.

#### Mean absolute error (MAE)

MAE measures the average absolute difference between the predicted ratings and the actual ratings for all items in the recommendation list.

11 $$\begin{aligned} \text {MAE} = \frac{1}{n} \sum _{i=1}^{n} |p_i - \widehat{p}_i| \end{aligned}$$

where,

n is the number of observations, $p_i$ is the actual value of the $i^{th}$ observation and $\widehat{p}_i$ is the predicted value of the $i^{th}$ observation.

#### Root mean square error (RMSE)

RMSE is another measure of the prediction error of a recommendation system.RMSE is similar to MAE but takes the square root of the average of the squared differences between predicted ratings and actual ratings.

12 $$\begin{aligned} \text {RMSE} = \sqrt{\frac{1}{n} \sum _{i=1}^{n} (p_i - \widehat{p}_i)^2} \end{aligned}$$

where,

n is the number of observations, $p_i$ is the actual value of the $i^{th}$ observation and $\widehat{p}_i$ is the predicted value of the $i^{th}$ observation.

## Results and discussion

This section presents a comparative analysis of classical and quantum-based correlation methods across four diverse datasets: Supermarket Sales, IMDB Top 250 Movies, MovieLens 10k, and BigBasket Product. The goal is to evaluate the effectiveness of the proposed IRQC approach, using key performance metrics such as Mean Absolute Error (MAE) and Root Mean Squared Error (RMSE).Table 2 provides correlation scores derived from both classical pearson, cosine and quantum method. The results highlight the improved ability of IRQC to detect meaningful item relationships.

**Table 2:** Retrieved correlation value along 5 recommendations.

**Dataset**	**Input**	**Output of Top N correlated item**		
		**Classical Pearson correlation with top correlated**	**Classical Cosine correlation with top correlated**	**IRQC with top correlated**
Supermarket Sales	Health and beauty	Food and beverages: 0.147704	Fashion accessories: 0.817437	Sports and travel: 0.8259
		Sports and travel: 0.030548	Food and beverages: 0.816699	Fashion accessories: 0.1902
		Electronic accessories : −0.019326	Electronic accessories: 0.802985	Home and lifestyle: 0.1030
		Home and lifestyle : −0.021837	Sports and travel: 0.797456	Electronic accessories: 0.0304
		Fashion accessories : −0.043420	Home and lifestyle: 0.786791	Food and beverages: −0.1732
BigBasket	Tamarind/Hunisehannu	Cleaning Cloth - Micro Fiber, G130018 : 1.0	Steel Lid/Cover For Utensils, Kadai & Tope - No.14: 1.0	Cream Of Tartar: 0.9988
		Basmati Rice/Basmati Akki - Sehat Mogra : 1.0	Chopping Board With Cutter & Dual Sided Blades - Small: 1.0	Grooming Solutions Kit: 0.9967
		Spunky and Funky Avatar Perfume : 1.0	Shake & Spray Serum : 1.0	Toilet Seat Sanitizer Deodoriser : 0.9931
		Coffee Sprinkles - Silky Hazelnut 1.0	Phenyl - Jasmine, Fresh: 1.0	Stainless Steel Tomato Knife - 8 Inch, Silver: 0.9927
		5 Meter Flexible Wire Link 1089 : 1.0	Ayurvedic Tea Masala - 21 Active Ingredients: 1.0	Face Shield A4 Size: 0.9922
IMDB Top 250 Movies	Ikiru	Amélie : 1.0	A Clockwork Orange: 1.0	The Green Mile: 0.9742
		Vertigo : 1.0	2001: A Space Odyssey : 1.0	Stand by Me: 0.9634
		Inglourious Basterds: 1.0	A Separation: 1.0	Batman Begins: 0.9333
		Lawrence of Arabia : 1.0	Amélie: 1.0	Logan: 0.8517
		To Kill a Mockingbird : 1.0	Citizen Kane: 1.0	Chinatown: 0.8252
MovieLens 10k	Heat (1995)	Philadelphia (1993) : 0.686060	Rock, The (1996) : 0.522755	Arthur 2 On the Rocks (1988): 0.9150
		Slumdog Millionaire (2008) : 0.662325	Twelve Monkeys (a.k.a. 12 Monkeys) (1995): 0.510677	Pompatus of Love, The (1996): 0.8831
		Léon: The Professional (a.k.a. The Professional) (Léon) (1994) : 0.651800	Léon: The Professional (a.k.a. The Professional) (Léon) (1994): 0.497140	Big Stan (2007): 0.8742
		Bourne Ultimatum, The (2007) : 0.611546	Casino (1995): 0.492802	Hope Floats (1998): 0.8709
		Aliens (1986) : 0.597448	Fargo (1996): 0.485918	Must Love Dogs (2005): 0.8602

For classical approaches, the perfect correlations are made up of sparse data or small overlapping ratings across products, where even a few matching entries with the same values are enough to create artificially high similarity scores. In contrast to classical correlation, which is plagued by instability on sparse datasets, the quantum correlation approach is stable because it uses entanglement-based encoding, which enables more accurate similarity estimation even with small user-item overlap. The results from the Supermarket Sales datasets revealed weak relationships between product categories, with most scores being close to zero or negative on the classical approach. The top correlation on the classical pearson approach is 0.147704, classical cosine is 0.817437, while IRQC identified a much stronger correlation of 0.8259. This suggests that IRQC can identify better relationships than the classical techniques, making it more sensitive to underlying consumer behavior patterns. In the BigBasket dataset, classical methods yielded perfect correlations of 1.0 for multiple items, providing no differentiation between them. IRQC has slight variability, with scores ranging from 0.9988 to 0.9922. This granularity suggests that IRQC can better distinguish relationships between highly correlated items, which may be useful in inventory analysis.For the IMDB Top 250 dataset, classical methods also produced 1.0 perfect correlations across all movie pairs. IRQC has not performed well for movie data, but in contrast revealed a more nuanced correlation structure. The MovieLens dataset showed moderate correlations of both classical methods, with a top score of 0.6860 and 0.522755. IRQC produced consistently higher correlations, such as 0.9150 as the top score for movie input data. These results highlight IRQC’s ability to obtain a high correlation, offering a richer understanding of user preferences on quantum model.

**Table 3:** Top-5 correlated items with similarity scores (80:20 split).

**Dataset**	**Input**	**Output of Top N correlated item for split 80:20**	
		**Classical Pearson correlation with top correlated**	**IRQC with top correlated**
Supermarket Sales	Health and beauty	Sports and travel: 0.542005	Electronic accessories: 0.6545
		Electronic accessories: 0.179028	Food and beverages: 0.4832
		Fashion accessories: 0.065036	Fashion accessories: 0.0150
		Food and beverages: −0.223439	Sports and travel: 0.0034
		Home and lifestyle: −0.333791	Home and lifestyle: −0.1886
BigBasket	Tamarind/Hunisehannu	Tea – Masala: 1.0	Spinach - Chopped: 0.9689
		Jowar Daliya: 1.0	Cashew/Godambi - Split: 0.9464
		Noodles - Chow Mein: 1.0	Organic - Almond: 0.9257
		Fragrance Body Spray- Good Morning : 1.0	Peppermint & Black Seed Lip Polish: 0.9251
		Fashion accessories : −0.043420	Food and beverages: −0.1732
IMDB Top 250 Movies	Ikiru	A Separation: 1.0	Logan: 0.9918
		Citizen Kane: 1.0	Monty Python and the Holy Grail: 0.9809
		Good Will Hunting: 1.0	Star Wars: Episode V - The Empire Strikes Back: 0.9779
		Eternal Sunshine of the Spotless Mind: 1.0	A Separation: 0.9525
		Amélie: 1.0	Gran Torino: 0.9521
MovieLens 10k	Heat (1995)	Slumdog Millionaire (2008): 0.822222	Outlaw Josey Wales, The (1976): 0.9920
		Philadelphia (1993): 0.802527	Down a Mountain, The (1995): 0.9909
		12 Angry Men (1957): 0.749979	Kill Bill: Vol. 2 (2004): 0.9874
		Aliens (1986): 0.706279	Tequila Sunrise (1988): 0.9670
		Harry Potter and the Half-Blood Prince (2009): 0.671984	Cabinet of Dr. Caligari, The (Cabinet des Dr. Caligari., Das) (1920): 0.9619

The findings in Table 3 demonstrate the top-5 correlated items achieved by classical Pearson correlation as well as the proposed IRQC method in an 80: 20 train/test split. Similarity computations are made only on the training set and the evaluation of recommendations on the test set is made to prevent data leakage. In all data sets, IRQC tends to generate a greater quantity and larger magnitude positive correlations than classical Pearson correlation. In the Supermarket Sales dataset where IRQC can give stronger associations like Electronic accessories (0.6545), as compared to the classical correlation which gives relatively lower values. Likewise, in the BigBasket and IMDB datasets, classical Pearson correlation reveals that there are a few perfect correlations (1.0), and it is possible that its results are due to overfitting or the sparsity effect, whereas IRQC provides more graded and discriminative similarity scores. In MovieLens 10k dataset, IRQC is reported to have a consistently higher correlation value, implying it is more efficient in tapping latent relationship between items. On the whole, these findings suggest that the suggested IRQC scheme has a more refined and strong representation of similarity when evaluated in an appropriate train/test scheme.

Certain product or movie pairs have values of classical correlation equal to 1.0, which can be explained as due to sparsity in the rating matrix as opposed to a real similarity semantically. Pearson correlation is ill-posed when the number of observations contained in both movies or products is very sparse, and approaches saturation at the two extremes. This bias is a known drawback of classical measures of correlation that is caused by sparsity. Conversely, the suggested Quantum Correlation is based on expectation values of entangled quantum states, and not on direct co-occurrence statistics, which leads to smooth and less saturated similarity estimates.

Although IRQC correlation values of BigBasket are in a high range which is rather narrow, distribution level analysis indicates that IRQC has more variance and more score separation among item pairs than classical correlation, which would tend to lies most of the similarities into very comparable values. This implies that at the high correlation values, there is indeed no longer a saturation point in IRQC but can indeed generate finer differentiations of similarities, which prove especially valuable when the correlations are then used to rank and respond to top-K recommendation tasks.

The classical methods of correlation require a quadratic increase in the correlation algorithm with the amount of items, but the quantum framework of correlation has a linear increase based on the observed amount of interactions. But it is limited in scalability to circuit depth, exponential growth of the state-space in simulators, the significance of shallow circuits and hybrid execution techniques to large-scale implementation.

**Fig. 3:**
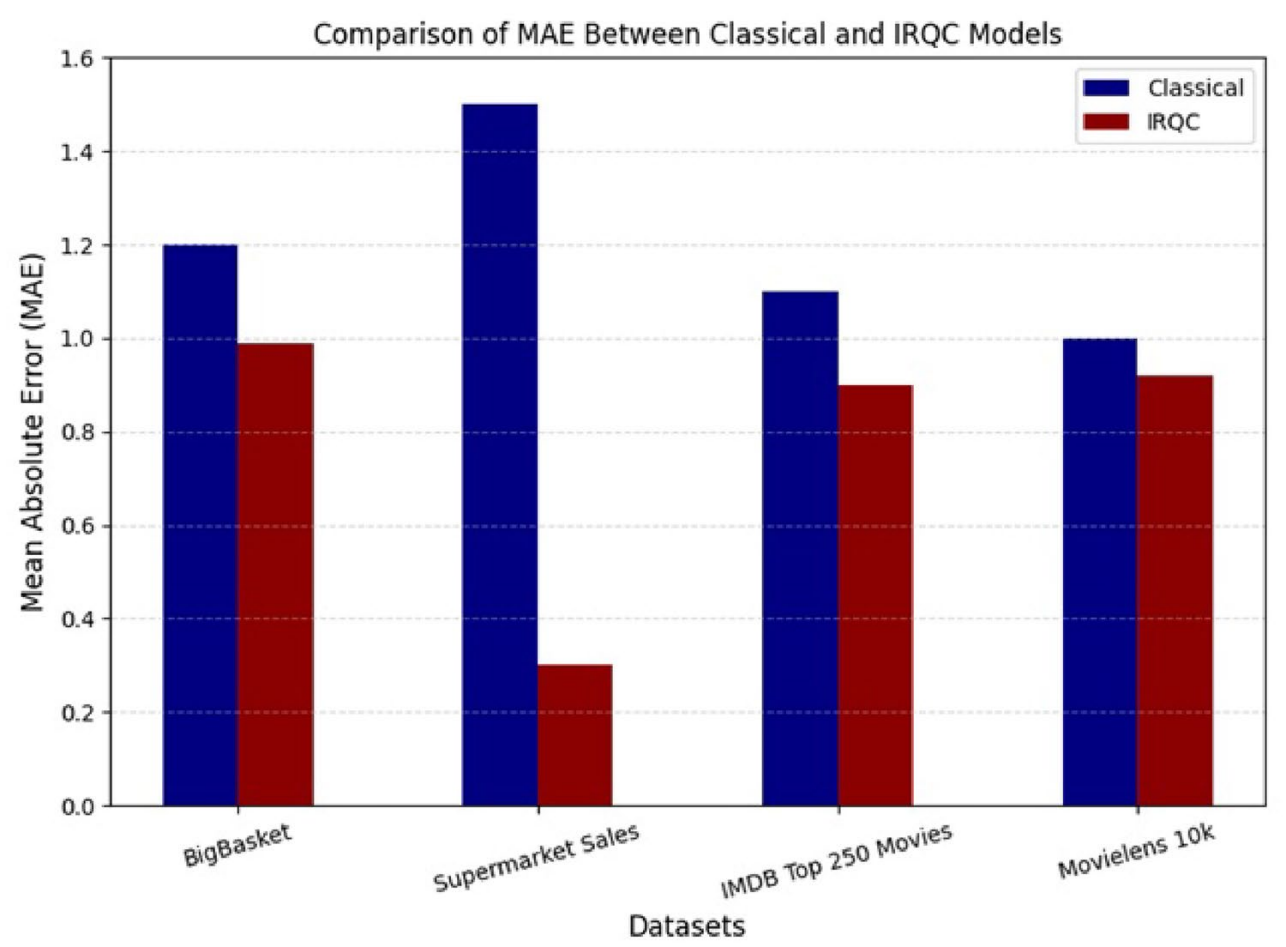
Comparison of Mean Absolute Error (MAE) between the classical correlation-based model and the proposed IRQC model on four datasets. The IRQC model demonstrates clear error reduction across all datasets, with a particularly pronounced improvement on the Supermarket Sales dataset, where the error is reduced dramatically compared to the classical method, demonstrating the effectiveness of the quantum correlation mechanism.

As shown in Figure 3, presents a comparative analysis of the Mean Absolute Error (MAE) values for the classical correlation-based model and the proposed IRQC model across four benchmark datasets: BigBasket, Supermarket Sales, IMDB Top 250 Movies, and MovieLens 10k. The X-axis represents the datasets, while the Y-axis denotes the MAE values(unitless), which measure the average magnitude of prediction errors irrespective of direction. Across all datasets, the IRQC model consistently achieves lower MAE values than the classical model, indicating enhanced prediction accuracy. The most substantial improvement is observed in the Supermarket Sales dataset, where the IRQC model results in a significant reduction in error. These results indicate that the proposed model delivers more accurate and stable predictions, particularly in datasets with higher price variability or domain-specific complexity.In the instance of“BigBasket,”IRQC model has a much lower MAE of 0.99, compared to classical correlation’s greater MAE of 1.20. Similarly, for“Supermarket Sales,”quantum correlation has a striking MAE of 0.30, but classical correlation has a significantly larger MAE of 1.48. In the case of the“IMDB Top 250 Movies,”IRQC model is still competitive, with an MAE of 0.90, whereas classical correlation has a slightly higher MAE of 1.1. Finally, for“MovieLens 10k,”item recommendation quantum correlation outperforms classical correlation with a significantly lower MAE of 0.92, compared to 1.0 for classical correlation. In summary, the results consistently indicate item recommendation and quantum correlation’s superiority, as it consistently produces lower MAE values across all datasets, indicating its potential to improve accuracy in predicting correlation when compared with traditional correlation.

**Fig. 4:**
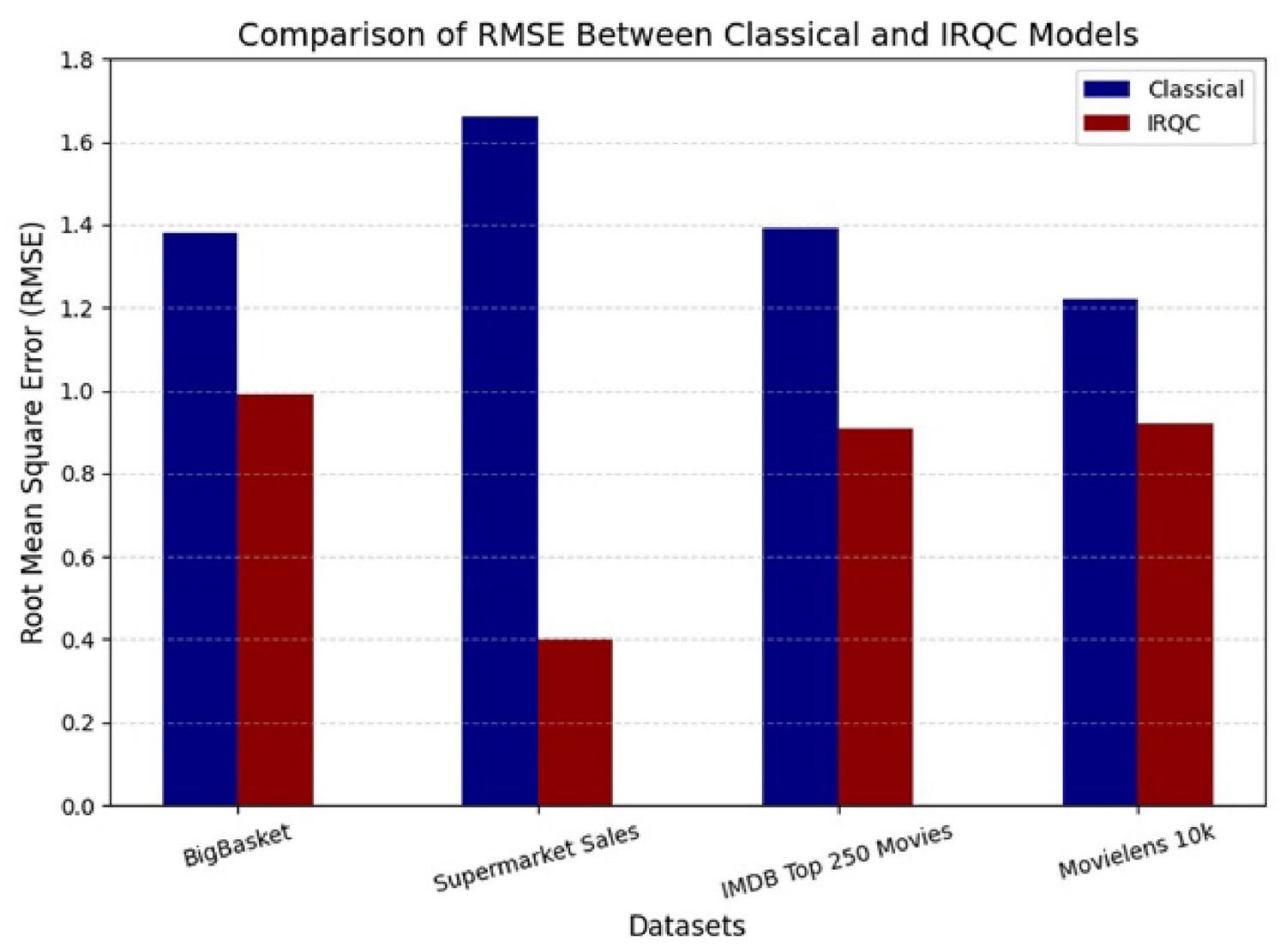
Comparison of Root Mean Square Error (RMSE) for classical and IRQC-based recommendation models on four benchmark datasets. The IRQC model demonstrates clear error reduction across all datasets, with the most pronounced improvement observed on the Supermarket Sales dataset, highlighting the effectiveness of the proposed quantum correlation framework in handling sparse and transactional data.

As shown in figure 4, presents a comparative analysis of the Root Mean Square Error (RMSE) values for the classical correlation-based model and the proposed IRQC model across four benchmark datasets: BigBasket, Supermarket Sales, IMDB Top 250 Movies, and MovieLens 10k. The X-axis represents the datasets, while the Y-axis indicates the RMSE values(unitless), which measure the square root of the mean squared prediction errors-thereby penalizing larger errors more heavily. The IRQC model consistently outperforms the classical model by achieving lower RMSE values across all datasets, reflecting not only improved average accuracy but also better handling of outliers and extreme deviations.IRQC often outperform their classical counterparts, as seen by significantly lower RMSE values. The classical correlation had an RMSE of 1.38 for the BigBasket dataset, but the IRQC model had a remarkably smaller RMSE of 0.99. A similar pattern was observed in the Supermarket Sales dataset, with the classical correlation yielding an RMSE of 1.66 and the item recommendation quantum correlation yielding a significantly better RMSE of 0.40. In the IMDB Top 250 Movies dataset, the classical correlation had an RMSE of 1.39, whereas the IRQC had an RMSE of 0.91. Finally, conventional correlation yielded an RMSE of 1.22 for the MovieLens 10k dataset, whereas quantum correlation provided an RMSE of 0.92. These findings show that proposed model have a strong advantage over classical correlations.

In IRQC, the target is the classical item-item correlation matrix based on the data (groundtruthmatrix) that acts as the reference or ground truth. No train/test split is used since the code calculates correlations between the whole dataset. The classical approach involves Pearson correlation of the products, whereas the quantum approach (IRQC) calculates correlations by the specified quantum circuit of every pair of products. Classical correlation ranking is then compared to the IRQC predicted correlations by ranking them and evaluating them using Recall@K and NDCG@K, and this allows an assessment of the ability of the quantum method to reproduce the relative similarity structure among products.

Although these findings are encouraging, there are some limitations that are essential for real-world applicability. It is computationally expensive and has high runtimes to simulate quantum circuits, particularly as qubit number and circuit depth increase. The classical computation of correlations is quadratic, and scales instantly, whereas the 5-qubit quantum calculation of correlations is more entanglement-based, and costs more, requiring a few more seconds based on size of dataset and circuit depth. Furthermore, implementing IRQC on existing NISQ hardware is restricted by variables like low numbers of qubits, decoherence, and gate fidelity. Similar to classical models, IRQC suffers from the cold-start situation since it relies on past user-item interactions to determine the correlations. Scalability continues to be an issue due to the explosion in resources needed for more complicated datasets and larger quantum circuits. These difficulties have been elaborated on to offer a balanced perspective of the model’s strengths and weaknesses, and to suggest investigating hybrid quantum-classical methods and scalable encoding strategies as potential areas of future research.

Table 4 demonstrates the results of up to six qubits. The results of the main experiments presented in figures three and four were obtained on a number of qubits (5), which is in line with the described setup. The six-qubit entries represent an extended analysis, which illustrates the impact of increasing quantum circuit depth, represented by the number of qubits (Q = 1 to 6), on the prediction performance of the proposed IRQC model across four datasets: Supermarket Sales, BigBasket, IMDB Top 250, and MovieLens 10k. The table presents both the Mean Absolute Error (MAE) and Root Mean Square Error (RMSE) for each level of quantum depth. Overall, the results indicate a consistent trend where an increase in the number of qubits leads to higher prediction accuracy, particularly in the Supermarket Sales and IMDB datasets. For instance, in the Supermarket Sales dataset, the MAE decreases from 0.63 at Q=1 to just 0.20 at Q=6, while the RMSE improves from 0.67 to 0.25, suggesting a significant enhancement in performance. A similar pattern is observed in the IMDB Top 250 dataset, where both MAE and RMSE gradually decline from 0.99 at Q=1 to 0.82 and 0.83, respectively, at Q=6. The MovieLens 10k dataset also shows improvements, with MAE reducing from 1.18 at Q=1 to 0.88 at Q=6. However, the BigBasket dataset demonstrates more stable error metrics across all qubit levels, with minimal variance in MAE and RMSE values. This indicates that increasing quantum depth has a lesser effect on this particular dataset, possibly due to the characteristics of the data or sensitivity to noise. These findings suggest that deeper quantum circuits (i.e., higher qubit counts) allow the model to capture more expressive patterns and complex relationships, thereby enhancing prediction performance, especially in datasets with intricate or rich rating distributions.

**Table 4:** MAE and RMSE values across different datasets for varying quantum depths (Q = 1 to 6). Lower values indicate better prediction performance.

Qubit(Q)	Supermarket Sales		BigBasket		IMDB Top 250		MovieLens 10k	
	MAE	RMSE	MAE	RMSE	MAE	RMSE	MAE	RMSE
1	0.63	0.67	0.99	0.99	0.99	0.99	1.18	1.25
2	0.58	0.62	0.99	0.99	0.98	0.98	1.02	1.02
3	0.51	0.56	0.99	0.99	0.94	0.95	0.96	0.97
4	0.44	0.52	0.99	0.99	0.91	0.92	0.93	0.94
5	0.30	0.40	0.99	0.99	0.90	0.91	0.92	0.92
6	0.20	0.25	0.98	0.98	0.82	0.83	0.88	0.91

The performance difference between the traditional correlation-based model and the IRQC model was measured through independent t-tests performed across four benchmark datasets: Supermarket Sales, BigBasket, IMDB Top 250 Movies, and MovieLens 10k. In order to ensure statistical robustness, the tests were conducted on correlation scores distributions across 10 experimental runs on each dataset. As indicated by Table 5, the classical model had statistically significant underperformance in the Supermarket Sales (t = −15.7383, p = 0.0001) and BigBasket (t = −12.3018, p = 0.0003) datasets but did not yield significant results in the IMDB (t = 1.0000, p = 0.3370) and MovieLens 10k(t = −1.1058, p = 0.2947) datasets. Conversely, the suggested IRQC model showed statistically significant and high performance gains in three datasets: BigBasket (t = 87.0338, p < 0.0001), IMDB (t = 22.0039, p < 0.0001), and MovieLens 10k (t = 16.6372, p = 0.0001). While the IRQC model did not show statistical significance for Supermarket Sales (t = 2.1369, p = 0.0994), the positive t-statistic reveals an increasing trend in performance. These results indicate that the IRQC model outperforms the traditional method at all times, especially in larger and more complicated datasets. The statistically significant gains confirm the promise of quantum-enhanced recommendation methods to provide more accurate and scalable solutions for recommendation systems in real-world applications.

**Table 5:** T-test Results Comparing Classical Correlation and Proposed IRQC Model.

**Dataset**	**Model**	**t-statistic**	**p-value**
Supermarket Sales	Classical	−15.7383	0.0001
	Proposed (IRQC)	2.1369	0.0994
BigBasket	Classical	−12.3018	0.0003
	Proposed (IRQC)	87.0338	0.0000
IMDB Top 250	Classical	1.0000	0.3370
	Proposed (IRQC)	22.0039	0.0000
MovieLens 10k	Classical	−1.1058	0.2947
	Proposed (IRQC)	16.6372	0.0001

The Table 6 compares the performance of the proposed IRQC model with classical Pearson correlation method on the widely used datasets of Supermarket Sales, BigBasket, IMDB Top 250 and MovieLens-10K on Recall@K and NDCG @K (K=2–5) has been tabulated. Recall@K estimates the model capability to recover the relevant items in the top-K recommendations, whereas the quality of the ranking is estimated by NDCG@K in terms of the more relevant items in the ranking. In all the datasets, the proposed IRQC produces better Recall@K values, which implies that more relevant items are retrieved, and the difference is stronger with the increase in K. Comparable or even better performance of IRQC is also observed in the terms of NDCG@K, especially at high K values, thus being better than maintaining the ranking quality with increasing the recommendation list. On the whole, these findings indicate that the suggested framework of IRQC is more effective than the classical method of correlation in terms of coverage of recommendations, as well as ranking.

**Table 6:** Performance comparison in terms of Recall@K and NDCG@K.

**Dataset**	**Models**	**Recall@K**				**NDCG@K**			
		**k=2**	**k=3**	**k=4**	**k=5**	**k=2**	**k=3**	**k=4**	**k=5**
Supermarket Sales	Classical Pearson Correlation	0.33	0.50	0.66	0.83	0.87	0.91	0.92	0.96
	Proposed IRQC	0.50	0.66	0.78	0.92	0.43	0.65	0.87	0.98
BigBasket	Classical Pearson Correlation	0.25	0.50	0.75	0.94	0.69	0.83	0.84	0.92
	Proposed IRQC	0.54	0.68	0.82	0.95	0.61	0.72	0.84	0.93
IMDB Top 250	Classical Pearson Correlation	0.40	0.52	0.74	0.91	0.40	0.60	0.80	0.94
	Proposed IRQC	0.53	0.64	0.80	0.93	0.52	0.65	0.78	0.95
MovieLens-10K	Classical Pearson Correlation	0.50	0.67	0.79	0.92	0.64	0.74	0.78	0.88
	Proposed IRQC	0.62	0.77	0.88	0.96	0.66	0.76	0.83	0.92

### Datasets used

The BigBasket, MovieLens, Supermarket Sales, and IMDb datasets were selected as representative of a broad set of recommendation situations, enabling us to test the performance and robustness of Quantum Correlations for diverse data scenarios. There are four different datasets for comparing classical correlation with proposed IRQC model to recommend items such as movies or products. The first dataset is the BigBasket dataset^36^, which is one of the largest Indian online food and grocery stores. This dataset consists of 27,555 items, 11 categories, 90 sub-categories, 426 product types, and 2,314 brands. The second Supermarket Sales dataset^37^ of products consists of 1000 instances and particularly contains sales information from three separate grocery locations over a period of three months, The third dataset covers the top 250 rated movies on IMDB as of 2021, offering a glimpse of the most popular and highly rated movies of recent times, and is part of the IMDB Top 250 Movies Database^38^, one of the biggest Indian online databases for movies and television series. The movie dataset consist of rating and genre for recommendation. The MovieLens 10 K dataset^39^comes from the MovieLens project, which is a movie recommendation service online. The information comprises movie ratings and other associated information submitted by the users of the MovieLens website^40^. The dataset contains 100,000 ratings from approximately 600 users on about 9,000 movies.The MovieLens 10k dataset was collected in the MovieLens repository, and under the title recommended for education and development, it was downloaded and the rest of the dataset was collected from the Kaggle repository.

### Experiment setup

The experiments were performed on a machine with an Intel i3 processor and 8 GB of RAM. All the implementations were done through Google Colab, where a Python notebook was established to execute the experiments. Necessary libraries like PennyLane, scikit-learn, matplotlib, pandas, and numpy were installed in order to facilitate both quantum and classical methods. To measure performance, the quantum correlation was compared with classical correlation methods (e.g., Pearson, **Spear**man) using error metrics like Mean Absolute Error (MAE) and Root Mean Squared Error (RMSE). This study assisted in evaluating the efficiency and usability of quantum algorithms in modeling product relationships in recommendation tasks.

## Conclusion

This study is aimed to examine the effectiveness of classical and quantum correlation approaches in boosting the accuracy of recommender systems across multiple datasets. The findings suggest that quantum correlations frequently outperform classical approaches, yielding much lower MAE and RMSE values. For the BigBasket dataset, the quantum technique has got an MAE of 0.99 compared to 1.20 for the conventional method, with similar improvements seen in the“Supermarket Sales,”“IMDB Top 250 Movies,” and“MovieLens 10k”datasets.These results emphasize the potential of quantum computing to better capture complicated interactions and increase recommendation accuracy, highlighting its promise for future applications in machine learning and recommender systems. Although the work yielded promising results, the use of quantum simulators may not accurately represent the difficulties in implementing quantum algorithms on actual hardware. Future research should focus on testing these methods on actual quantum devices to measure scalability and performance. Additionally, hybrid quantum classical techniques should be studied to utilize the strengths of both paradigms, enabling more realistic solutions for large-scale applications.

## Data Availability

https://www.kaggle.com/datasets/chinmayshanbhag/big-basket-products, https://www.kaggle.com/datasets/faresashraf1001/supermarket-sales, https://www.kaggle.com/datasets/rajugc/imdb-top-250-movies-dataset, https://grouplens.org/datasets/movielens/.
